# Case Report: Migraine-Like Headache With Visual Aura Initiated by Endovascular Coiling Treatment for a Posterior Cerebral Artery Aneurysm

**DOI:** 10.3389/fneur.2021.646029

**Published:** 2021-03-17

**Authors:** Xin Chen, Juan Zhang, Han-Li Li, Zi-Ru Deng, Long Wang, Li Cao, Cheng-Juan Xie, Yu Wang

**Affiliations:** ^1^Department of Neurology, Epilepsy and Headache Group, The First Affiliated Hospital of Anhui Medical University, Hefei, China; ^2^Department of Neurology, The Fourth Affiliated Hospital of Anhui Medical University, Hefei, China; ^3^Department of Electrocardiogram, The First Affiliated Hospital of Anhui Medical University, Hefei, China

**Keywords:** case report, endovascular therapy, migraine, posterior cerebral artery aneurysm, visual aura

## Abstract

Cervical, anterior, and middle cerebral artery aneurysm is a causative factor for migraine, and endovascular treatment usually improves migraine headache. Posterior cerebral artery (PCA) aneurysm is a rare condition, and its association with migraine is very rarely reported. In addition, endovascular coiling treatment causing migraine-like headache has never been reported. Here, we describe a newly developed migraine-like headache with visual aura after endovascular coiling treatment for PCA aneurysm in a 31-year-old female patient. One month after the endovascular therapy, the patient stopped using the antiplatelet agents clopidogrel and aspirin and presented with an episodic headache attack twice a month with typical migraine features, including visual aura, right-sided temporal throbbing pain accompanied with nausea, vomiting, and photophobia. The recurrence of migraine-like headache with visual aura was terminated by clopidogrel administration. The generation of the migraine-like headache with visual aura is probably associated with microemboli due to endovascular coiling. This case supports the hypothesis that migraine with aura can be associated with microemboli of variant origins.

## Background and Importance

Migraine-like headache, or symptomatic migraine, has often been reported to be caused by pathological alterations affecting migraine pain-related structures of the brain, such as brainstem and cerebral hemisphere (1–3). Although migraine has long been accepted to be a brain hyperexcitability disease (4), brain vascular alterations are continually reported to be causative factors for the onset of recurrent headaches meeting the diagnostic criteria of migraine by presentation. For example, moyamoya disease has been recognized to be the cause of migraine-like headaches for long time (5), and carotid-cavernous fistula and dissections of intracranial and cervical arteries are reported to be the cause of migraine-like headaches (6, 7). Another form of brain vascular alteration, cervical, anterior, and middle cerebral artery aneurysm, is also demonstrated to be associated with increased prevalence of migraine headaches with or without aura (8), and surgical treatment with either coil embolization or clipping relieved the recurrent headaches (9–11), indicating that cranial aneurysm is a causative factor of migraine. Posterior cerebral artery (PCA) aneurysm is a rare condition, and its association with migraine is rarely reported. Here, we report on a patient with PCA aneurysm who developed migraine-like headaches with visual aura after endovascular coiling treatment. To the best of our knowledge, this is the first report that a treatment with endovascular coiling of a PCA aneurysm initiated a migraine-like headache with visual aura.

## Case Presentation

**Figure 1:**
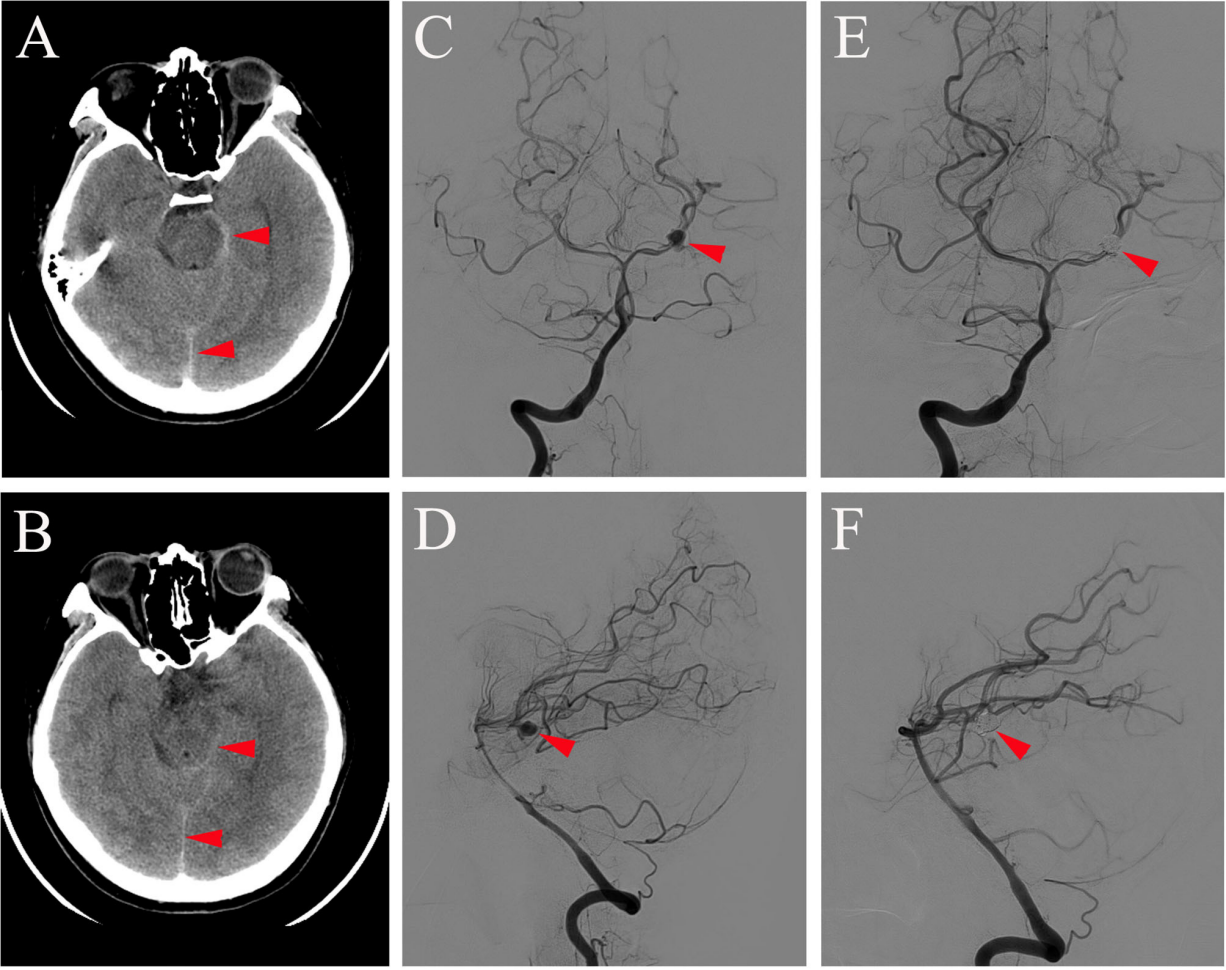
Brain CT **(A,B)** conducted on the day of symptom onset showing subarachnoid hemorrhage (red arrowhead on **A,B**) and DSA of antero-posterior view **(C,E)** and lateral view **(D,F)** obtained 7 days after symptom onset showing aneurysm at the P1-P2 segment of the left PCA before (red arrowhead on **C,D**) and after coil embolization (red arrowhead on **E,F**).

A 31-year-old woman came to our department with an abrupt and severe headache for half a day. At the time of admission, her Fisher scale was on grade 2 and the Hunt and Hess scale was on grade 1. She had no motor or sensory deficit but had signs of meningeal irritation. There was no history of trauma, hypertension, diabetes, or other disease. Computed tomography (CT) of the brain was conducted immediately and showed a subarachnoid hemorrhage (SAH) (Figures 1A,B). Thus, the patient was diagnosed with SAH and accepted regular treatment with intravenous mannitol and nimodipine, and the headache was greatly relieved. Seven days after headache symptom onset, the patient underwent digital subtraction angiography (DSA) examination under general anesthesia, and the result showed the presence of an aneurysm of the left PCA at the P1-P2 segments, measuring 4 × 5 mm with a neck of 1.5 mm (Figures 1C,D). Then, the patient underwent stent-assisted coil embolization of the aneurysm (Figures 1E,F). Stent-assisted coil embolization did not cause any neurological complication, and the patient's headache disappeared gradually within days postembolization. One week after coil embolization was conducted, brain CT examination was reperformed, and the result showed no abnormality in brain parenchyma. The patient was discharged and required to continue treatment with oral antiplatelet agents aspirin (100 mg/day) and clopidogrel (75 mg/day), which started the day after endovascular coiling treatment.

**Figure 2:**
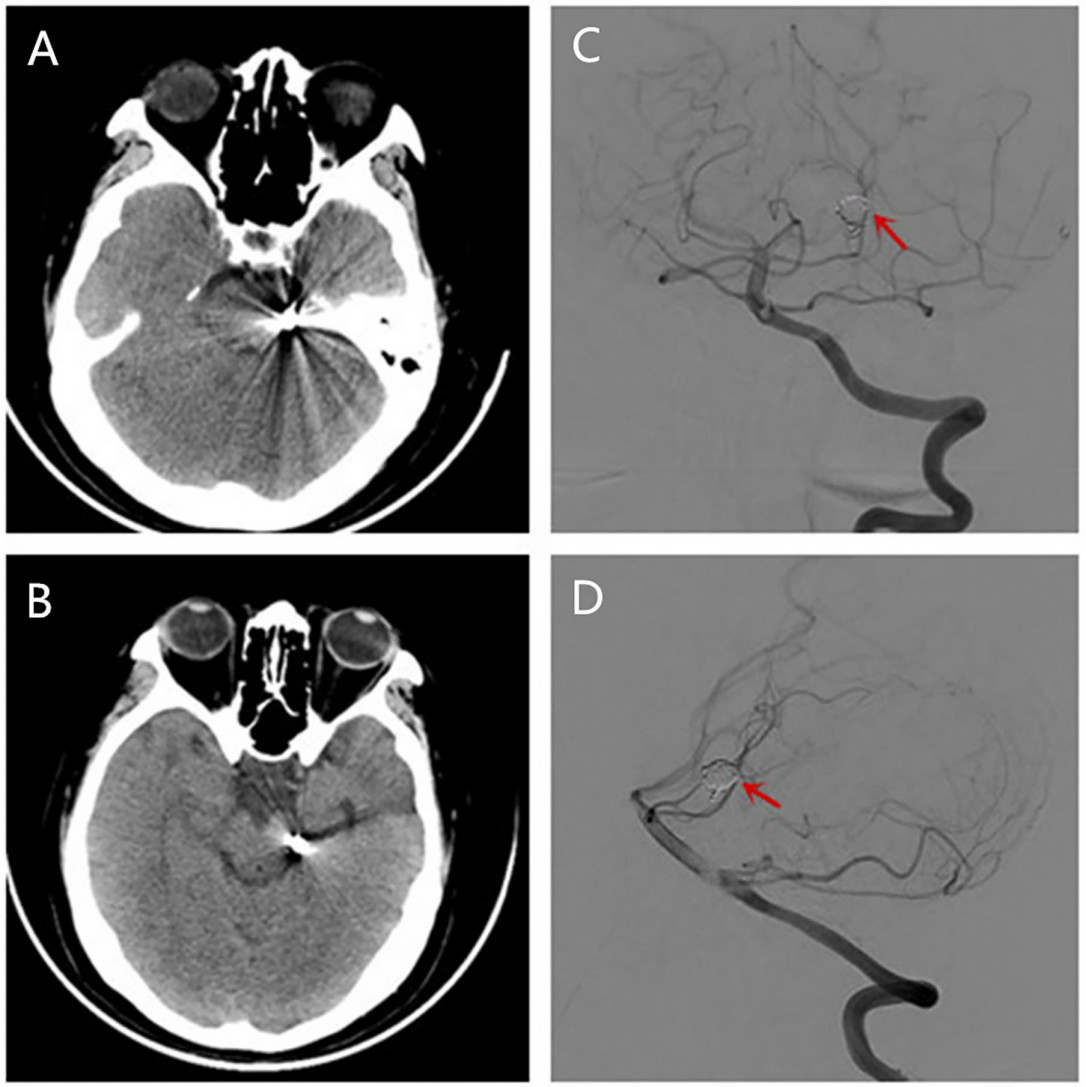
Brain CT **(A,B)** conducted showing no localized hemorrhage or ischemic lesions after she had a migraine-like headache. Reexamination of DSA showing the coil was stable and there was no *in situ* thrombus **(C,D)**.

**Figure 3:**
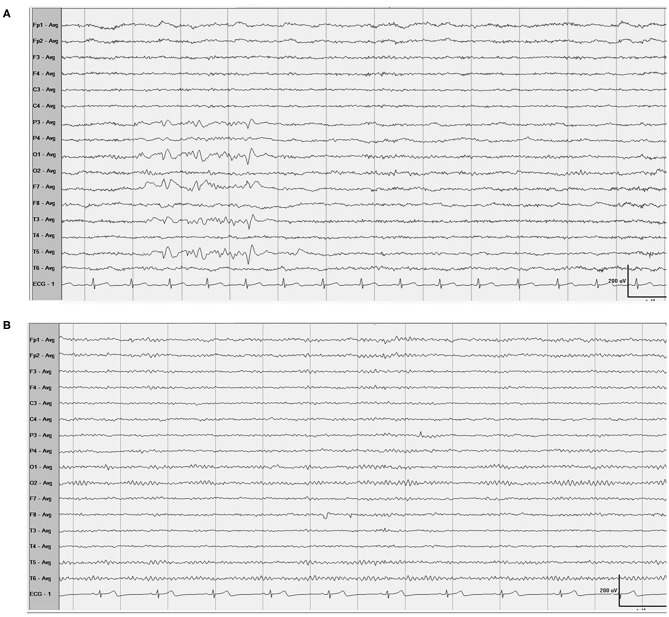
EEG **(A)** Showed spike-and-slow wave complexes in the left occipital and temporal areas, and 1 month after the readministration with clopidogrel, EEG **(B)** showed normal.

One month after endovascular therapy, aspirin, and clopidogrel were abandoned by the patient due to gingival bleeding and skin ecchymosis. On the day the antiplatelet agents were halted, the patient presented with transient visual disturbances characterized by spotted flashes around the bilateral visual field and a water ripple-like image stemming from the center of the bilateral visual field and spreading around. This visual disturbance lasted for 7–8 min and was then succeeded by severe right-sided throbbing headache lasting for about 2–3 h. The headache was accompanied with nausea, vomiting, and photophobia and aggravated by head movement. Head CT was performed immediately showing no localized hemorrhage or ischemic lesions (Figures 2A,B). The patient was underwent DSA examination again, showing that the coil was stable and there was no *in situ* thrombus (Figures 2C,D). From then on, she experienced episodic visual disturbance and headache attacks similar to the episode on the day the antiplatelet agents were halted every month with a frequency of two times a month. Each episode of headache would last 2–3 h. The headache was moderate to severe in severity and throbbing and pressure-like in character. Photophobia and nausea accompanied when the headache was severe. Before this event, she had neither a personal nor familial history of recurrent headaches. This recurrent event was considered to be either migraine with aura or epilepsy, and an electroencephalography (EEG) examination was conducted. The EEG showed epileptic discharges in the left occipital and temporal areas. A treatment with sodium valproate 500 mg twice a day was chosen for the patient as sodium valproate can usually effectively and prophylatically treat either migraine or epilepsy. The episodic visual aura and succedent headache were still recurring twice or even more times a month during the 3 months of sodium valproate treatment, but the headache severity seemed reduced. Sixteen months after endovascular coiling treatment, the patient accepted EEG examination again in our department, and spike-and-slow wave complexes could be detected in the left occipital and temporal areas (Figure 3A). The patient reaccepted antiplatelet treatment with clopidogrel (75 mg/day), omitting valproate and aspirin, and her migraine-like headache with visual aura relieved during the 4 months of follow up. One month after the readministration with clopidogrel, EEG was reconducted, and neither epileptiform discharges nor asymmetric background activity was observed (Figure 3B).

## Discussion

According to the diagnostic criteria of the International Classification of Headache Disorders, 3rd edition (beta version) (12), this patient's headache with precedent visual aura may be classified as headache attributed to cranial or cervical vascular disorder but not meeting the criteria for any of its subclass. The subclass of headache attributed to carotid or vertebral angioplasty is most closely related to the headache symptom of our patient but does not cover it (Table 1) (2). The recurrent attacks of unilateral throbbing headache, photophobia, nausea, and precedent visual aura are consistent with a migraine-like phenotype with visual aura, i.e., migraine-like headache with visual aura although not meeting formal diagnostic criteria for migraine with aura. On the other hand, the clinical presentation of the left occipital and temporal spikes on EEG drive us to consider a possible diagnosis of epilepsy as patients accepted coiling treatment for ruptured cranial aneurysm have a seizure or epilepsy incidence of 11.1% from the literature (13), and the blood supply of the occipital and temporal areas is from PCA. However, the antiplatelet treatment relieved the head pain and visual aura, indicating that the recurrent headaches and the precedent visual symptoms were not a presentation of epilepsy. Our patient had neither a personal nor familial history of recurrent headache, and her migraine-like headache with visual aura occurred after endovascular therapy. Between endovascular therapy and occurrence of the episodic headache event, there was an interval of 1 month during which the patient was taking antiplatelet agents, and the recurrent headache with visual aura occurred the day after antiplatelet agents were abandoned. This logical relationship indicates that the migraine-like headache with visual aura was caused by endovascular therapy, and antiplatelet treatment gave a remission of the recurrent events. On the other hand, causative association of SAH but not of endovascular therapy should also be considered as focal SAH can cause a clinical presentation of repetitive migraine auras due to hemorrhagic stimulation to the occipital cortex, but the repetitive events are transient (14). Thus, the long-term recurrence of migraine-like headache with visual aura in our patient was not due to the SAH itself. Other headaches that commonly happen shortly after or during the endovascular treatment procedure are also transient, lasting for <2 h and usually about 10 min although some were unilateral and throbbing in character. They were not recurrent and never accompanied with visual aura (15, 16). Thus, the phenotype of headache shortly after endovascular therapy is different from the headache event in our patient.

**Table 1:** International Classification of Headache Disorders Third Edition (ICHD-3) criteria for headache attributed to carotid or vertebral angioplasty.

A	Any new headache, fulfilling criterion C	
B	Carotid or vertebral angioplasty has been performed	
C	Evidence of causation demonstrated by all of the following:	
	1	Headache has developed within 1 week of the angioplasty
	2	Headache has resolved within 1 month after the angioplasty
	3	Headache is on the same side as the angioplasty
D	Not better accounted for by another ICHD-3 diagnosis, and arterial dissection has been excluded by appropriate investigations	

A higher prevalence of migraine headaches with or without aura in patients with cranial aneurysm (8) and improvement of the preexisting migraine headache after endovascular treatment with either coiling or clipping (17) indicate that cranial aneurysm is a causative factor for migraine. Cranial aneurysms may be saccular, fusiform, or dissecting and can be located at various segments of the cranial artery. Most of the literature reports that migraine-associated aneurysms are saccular in shape. Cranial dissecting aneurysm is very rarely associated with migraine; there is one report showing that a dissecting aneurysm on MCA initiated migraine-like headache (18), another report showing that a dissecting aneurysm on PCA initiated migraine-like headache with visual defect (19), and a few reports showing that dissecting aneurysm on vertebral or anterior artery are closely associated with the onset of headache but not a type of migraine (20–22). These literature reports show that cranial aneurysm is either a causative or a facilitating factor for migraine attacks. Our patient had new onset of migraine-like headaches after endovascular treatment for aneurysm, indicating that the headaches were caused by the endovascular treatment but not by the aneurysm. Our case seems in disagreement with that of the literature reports.

In our patient, the aneurysm was located on the segment of PCA that supplies part of the temporal cortex, the calcarine, and the occipital cortex as well as parts of the brainstem and thalamus. The phenomenon that antiplatelet treatment caused an abortion of recurrent migraine-like headaches in our case indicates that the occurrence of migraine-like headache was associated with the stenting coil equipment. Thus, it might be proposed that microemboli generated from the coiling equipment migrated along the PCA into the occipital area, causing the visual aura and subsequent headache. This speculation is supported by an animal study in which microemboli were generated from the patent foramen ovale (PFO) and moved to the brain cortex and induced cortical spreading depression (CSD), the presumed basis of migraine aura and headache in patients with PFO (23). This is supported by a case report in which microembolic signals under transcranial doppler monitoring were repeatedly observed in the basilar artery, and the microembolic signals together with the migraine with aura symptoms disappeared simultaneously with removal of the PCA pseudoaneurysm (24). This microembolus-induced CSD is proposed to be the underlying pathophysiological mechanism of the association between migraine and many cardiac diseases, such as atrial fibrillation (25), cardiac myxoma, PFO, atrial septal defect (ASD), atrial septal aneurysm, mitral valve prolapse, and congenital heart disease (26). The preventive effect of the antiplatelet on migraine-like headache with visual aura caused by coiling in our patient is consistent with the prophylactic role of aspirin and clopidogrel, ticagrelor in some migraine patients with PFO (27, 28).

Given that the microemboli mechanism for the migraine headache attacks is correct, both the cranial aneurysm and ebdovascular coil embolization may cause generation of microemboli initiating migraine-like headache attacks. A cranial aneurysm may generate microemboli due to hemodynamic changes, and endovascular coil embolization may also generate microemboli due to disturbances of blood coagulation. Based on this theory, there is no discrepancy between literature reports that cranial aneurysm is either a causative or a facilitating factor for migraine attacks and our case presentation that endovascular coiling treatment for aneurysm causes recurrent migraine-like headache attacks.

## Conclusion

We demonstrate a rare case of migraine-like headache with visual aura triggered by endovascular therapy of PCA aneurysm. The generation of the migraine-like headache with visual aura is probably associated with microemboli due to the endovascular stenting coil. This case supports the hypothesis that migraine can be associated with microemboli of variant origins. Finally, from this case, we learned that we need to keep in mind that a secondary migraine with aura can be caused by endovascular coiling treatment and an antiplatelet agent may relieve the headaches.

## Data Availability Statement

The original contributions presented in the study are included in the article/supplementary material, further inquiries can be directed to the corresponding author/s.

## Ethics Statement

Written informed consent was obtained from the individual(s) for the publication of any potentially identifiable images or data included in this article.

## Author Contributions

XC and YW carried out the conception, treatment, and follow up. XC drafted the manuscript. JZ and C-JX were involved in the manuscript drafting. H-LL, Z-RD, and LC were involved in the interpretation of data. YW revised the manuscript. All authors read and approved the final version of the manuscript.

## Conflict of Interest

The authors declare that the research was conducted in the absence of any commercial or financial relationships that could be construed as a potential conflict of interest.
